# Association between dietary inflammatory index and fecal incontinence in American adults: a cross-sectional study from NHANES 2005–2010

**DOI:** 10.3389/fnut.2024.1364835

**Published:** 2024-07-15

**Authors:** Zhigang Li, Xing Chen, Jiaobao Huang, Fei Cheng, Zhao Wu, Lebin Yuan, Xiaodong Li, Wei Shen

**Affiliations:** Department of Gastrointestinal Surgery, The Second Affiliated Hospital of Nanchang University, Nanchang, China

**Keywords:** dietary inflammatory index, fecal incontinence, NHANES, association, dietary inflammatory potential

## Abstract

**Objective:**

Recent studies have demonstrated that the Dietary Inflammatory Index (DII) is relevant to abnormal gut health. However, there is a lack of studies that have explicitly explored the link between fecal incontinence (FI) and DII. The current study aims to explore the relationship between DII and FI.

**Methods:**

The cross-sectional study enrolled a total of 11,747 participants aged 20–85 from NHANES 2005–2010. Weighted logistic regression was conducted to evaluate the relationship between DII and FI, and restricted cubic spline (RCS) was employed to assess the dose-response relationship between DII and FI. Subgroup analyses were performed according to age, gender, race, and BMI.

**Result:**

DII levels were found to be significantly higher in patients with FI than in the normal population (*p* = 0.016). After adjusting for all covariates, DII was found to be significantly correlated with FI (model 2: Q4 vs. Q1, OR = 1.49, 95% CI: 1.04–2.14, *p* = 0.032, *p* for trend = 0.039). The dose-response curve revealed that there was no non-linear correlation between DII and FI (*p*-non-linear = 0.234). Subsequent subgroup analyses uncovered that DII was notably associated with FI in the old (Q4 vs. Q1, OR = 1.84, 95% CI: 1.07–3.18, *p* = 0.030), female (Q4 vs. Q1: OR = 2.02, 95% CI: 1.23–3.33, *p* = 0.008), non-Hispanic white (Q4 vs. Q1: OR = 1.70, 95% CI: 1.12–2.59, *p* = 0.015) populations.

**Conclusion:**

DII was positively associated with FI, particularly among old, female and non-Hispanic white individuals. Decreasing daily dietary inflammatory levels may be an effective tactic to prevent FI, but the precise mechanisms need to be further investigated.

## Introduction

Fecal incontinence (FI) is a very common gastrointestinal disorder, manifesting as symptoms like involuntary or uncontrollable bowel movements. Epidemiological research indicates that FI occurs in ~1 in 12 (8.3%) people worldwide, with higher prevalence in older (9.3%) and female (9.1%) populations (1). In addition to severely impairing quality of life (2), it's pertinent to highlight that FI has been identified as a contributor to cancers such as gastrointestinal tumor and lymphoma, depression, and sarcopenia (3–5). Therefore, it is crucial to identify the risk factors associated with FI and prevent its occurrence.

Dietary patterns have been evidenced to be implicated in the development of FI in previous studies. For instance, an earlier prospective study found that the FI group consumed more carbohydrates, manganese, and vitamin B1 than the normal group (6), hinting at a possible dietary influence on FI. In addition, the intake of certain dietary fibers such as psyllium reduces the risk of FI (7, 8), and reducing the consumption of a diet rich in fermentable oligosaccharides, disaccharides, monosaccharides, and polyols (FODMAP) has been proven to improve FI symptoms (9, 10). Recently, a prospective cohort study uncovered a significant relationship between pro-inflammatory diet and FI in older women (11), indicating a potential dietary inflammation implication on FI. Nevertheless, the relationship between dietary inflammatory levels and FI among the general population remains uncertain.

Dietary Inflammation Index (DII) is a new method for evaluating dietary inflammatory levels that is based on literature sources and considers the entire diet (consisting of 45 foods) rather than single nutrients or foods. It is suitable for assessing dietary inflammation levels in any patient with complete dietary data (12). To date, DII has been confirmed to be closely linked to various conditions, including cancer, cardiovascular disease, and sarcopenia (13–16). Regarding gastrointestinal diseases, Dong et al. found that DII was positively correlated with the likelihood of esophageal precancerous lesions (EPL) among high-risk esophageal squamous cell carcinoma (ESCC) patients in the Chinese population (17). A multicenter case-control study in Brazil suggested that a diet high in DII was linked to an increased risk of gastric adenocarcinoma (18). In addition, DII was found to be linked to a higher risk of developing colorectal adenomatous polyps (19) and Helicobacter pylori infection (20). Furthermore, recent studies have shown that DII is associated with chronic diarrhea, chronic constipation, and inflammatory bowel disease (21–23). However, limited research has been conducted on the association between DII and FI. We hypothesize that a high level of DII is significantly associated with FI. To this end, the aim of this study was to assess the relationship between DII and FI using the NHANES database.

## Methods

### Study design

NHANES is a large database maintained by the National Center for Health Statistics (NCHS) of the Centers for Disease Control and Prevention (CDC) to assess the health and nutritional status of adults and children in the United States. The project conducts health and nutrition surveys on a 2-year cycle using complex multistage probability sampling of representative populations from every county in the United States. The data for this study comes from the 2005–2006, 2007–2008, and 2009–2010 NHANES survey cycles, all of which are publicly available and can be accessed on the official NHANES website [NHANES–National Health and Nutrition Examination Survey Homepage (cdc.gov)]. We chose these three data cycles because only during that period did the Bowel Health Questionnaire (BHQ) detail FI.

**Figure 1:**
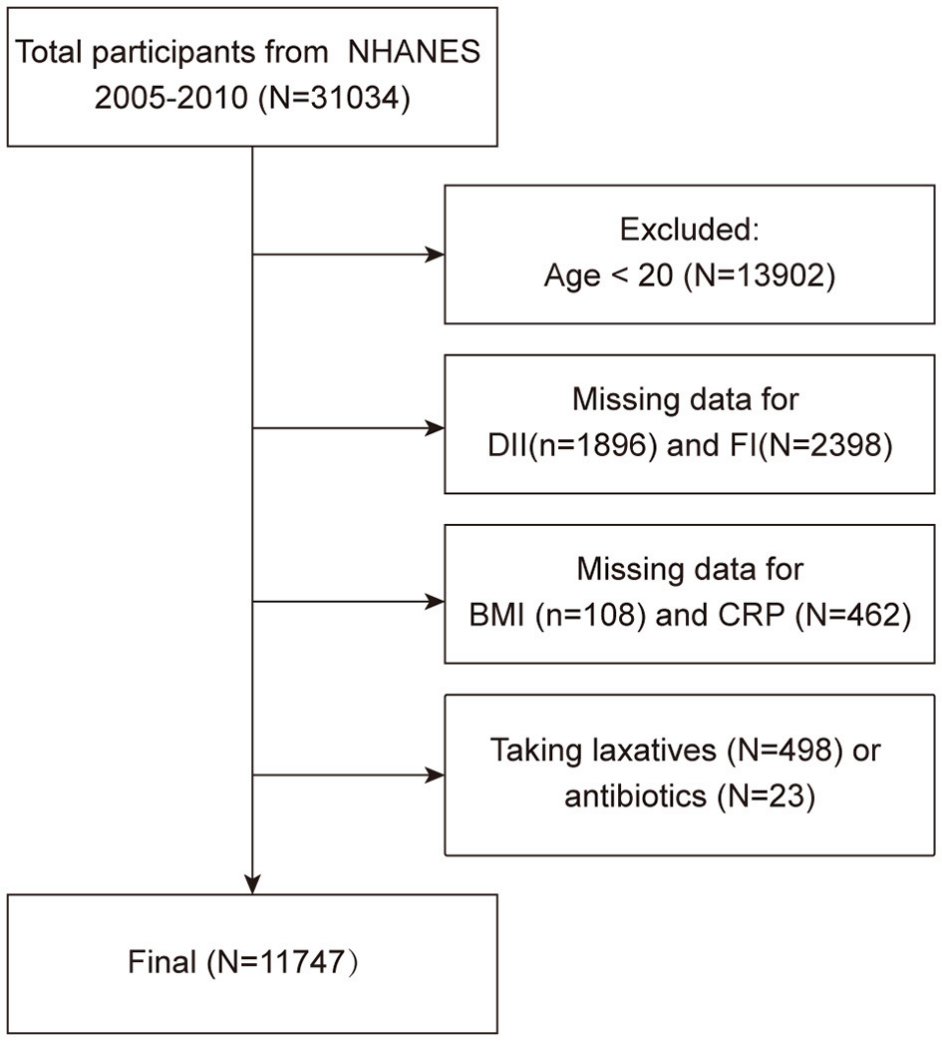
The flowchart of sample selection.

The exclusion criteria of the study were as follows (Figure 1): (1) aged < 20 years old (*n* = 13,902); (2) missing data for DII (*n* = 1,896) and FI (*n* = 2,398); (3) missing data for covariates: BMI (*n* = 108), CRP (*n* = 462); (4) individuals taking laxatives (*n* = 498) and antibiotics (*n* = 23). After eliminating the above participants, a total of 11,747 participants were finally enrolled in this cross-sectional study.

### Definition of FI

Data from the NHANES 2005–2010 BHQ questionnaire provides detailed personal interview data on FI and bowel function in adults aged 20 years and older. In the mobile examination vehicle (MEC), participants were questioned, “How often during the past 30 days have you had any amount of accidental bowel leakage that consisted of the four types of stool mentioned?” and “Bowel leakage consisted of gas, mucus, liquid or solid?” In addition, participants were provided with Bristol Stool Form Scale (BSFS) cards to assess stool form by answering the question “Please look at this card and tell me the number that corresponds to your usual or most common stool type.” Participants who were absent from the bowel health section of the interview resulting in missing data of FI were excluded. In the current study, FI was defined as at least 1 involuntary evacuation of solid, mucus, or liquid stools in a month, while gas leakage was not included.

### Calculation of DII

As an exposure factor in this study, DII was calculated by recording participants' dietary data over a 2-day period through a 24-h dietary recall interview within the MEC. Participants who were absent from the dietary recall interview were excluded. The formula for calculating the DII is as follows: DII for each nutrient or dietary ingredient = [(daily intake of the nutrient or dietary ingredient–global per capita daily intake of the nutrient or dietary ingredient)/standard deviation of the global per capita daily intake of the nutrient or dietary ingredient] ^*^ Inflammatory Effect Index (IEI) for the nutrient or dietary ingredient, and the sum of the DIIs for each nutrient or dietary ingredient will be the individual study subject's total DII score (12). In our study, we calculated DII from 27 of the 45 foods using “dietaryindex” R package developed by Zhan et al., which has been demonstrated to be in line with standard algorithms for calculating DII scores (24). Additionally, we categorized DII by quartiles for further analysis, in addition to the statistical analysis of continuous DII.

### Assessment of covariates

Based on previous studies, we identified several key variables that may affect fecal incontinence and included them as confounders in this study (11, 25–28). These variables include demographic characteristics such as age [young (20–44)/middle-aged (45–64)/old (65–85)], sex (male/female), race (non-Hispanic white/non-Hispanic black/Mexican American/other race), educational level (less than high school/high school/college and above), and ratio of family income to poverty (PIR) (< 2/≥2/unknow), as well as BMI [normal (< 25 kg/m^2^)/overweight (25–30 kg/m^2^)/obesity (≥30 kg/m^2^)], chronic diseases such as hypertension, diabetes, and hyperlipidemia, lifestyle habits such as smoking status, alcohol consumption, and physical activity, CRP (mg/dL), and total energy intake (kcal).

Smoking status was defined as follows: no-smoking, fewer than 12 cigarettes in their lifetime; past smoking, more than 12 cigarettes in their lifetime but not currently smoking; current smoking, more than 12 cigarettes in their lifetime and still smoking. Alcohol consumption was defined as follows: < 4 drinks in the past 12 months was defined as no or light drinking; more than 4 but < 12 drinks in the past 12 months was defined as moderate drinking; and 12 or more drinks in the past 12 months was defined as heavy drinking. Physical activity was defined as follows: when participants were asked, “Do you have a large increase in breathing or heart rate from vigorous exercise, fitness, or recreational activities, such as running or playing basketball for at least 10 min straight?” patients who answered “yes” to the question were defined as vigorous activity; when participants were asked, “Do you do any moderate-intensity exercise, fitness, or recreational activity that results in a small increase in respiration or heart rate, such as brisk walking, biking, swimming, or golfing, for at least 10 consecutive minutes?” patients who answered “yes” were defined as moderate. The remaining participants were classified as inactive in their daily activities. Participants were considered to have hyperlipidemia if they had total cholesterol≥200 mg/dL or triglycerides≥150 mg/dL or LDL≥130 mg/dL, or if they were diagnosed with hypercholesterolemia by another doctor or health professional. Hypertension was defined as having been told by a doctor that you have high blood pressure, prescribed medication for high blood pressure, or are currently taking antihypertensive medication. Diabetes was defined as having a history of diabetes as told by a doctor, insulin, or having a history of hypoglycemic medication.

### Statistical analysis

As recommended by the NHANES survey, appropriate weight was calculated for statistical analysis in this study. Continuous variables were presented as weighted means (±standard error, SE), and categorical variables were presented as unweighted counts (weighted percentages). Comparison between continuous variables was conducted using weighted *t*-test, while categorical variables were tested using weighted chi-squared test. In addition, participants were divided into four groups according to DII quartiles: Q1, < 0.47 (*n* = 2,942); Q2, 0.47–1.68 (*n* = 2,934); Q3, 1.69–2.71 (*n* = 2,960); Q4, >2.71 (*n* = 2,911).

Weighted logistic regression was used to assess the relationship between DII and FI, and three graded incremental logistic models were constructed: crude model was unadjusted; model 1 was adjusted for sex, age, race, education level, and PIR; model 2 built on model 1 by incorporating BMI, smoking status, alcohol consumption, physical activity, hyperlipidemia, hypertension, and diabetes, CRP, and energy intake. Restricted cubic spline plot (RCS) was used to evaluate the non-linear relationship between DII and FI. Finally, subgroup analyses were performed based on age, sex, race, and BMI. Benjamini-Hochberg method of multiple testing was utilized to correct the *p*-values.

All statistical analyses were conducted using R (version 4.3.2), and weighted analyses were performed using the “survey” package. A two-tailed *p*-value < 0.05 was considered statistically significant.

## Result

### Baseline characteristics of selected participants

**Figure 2:**
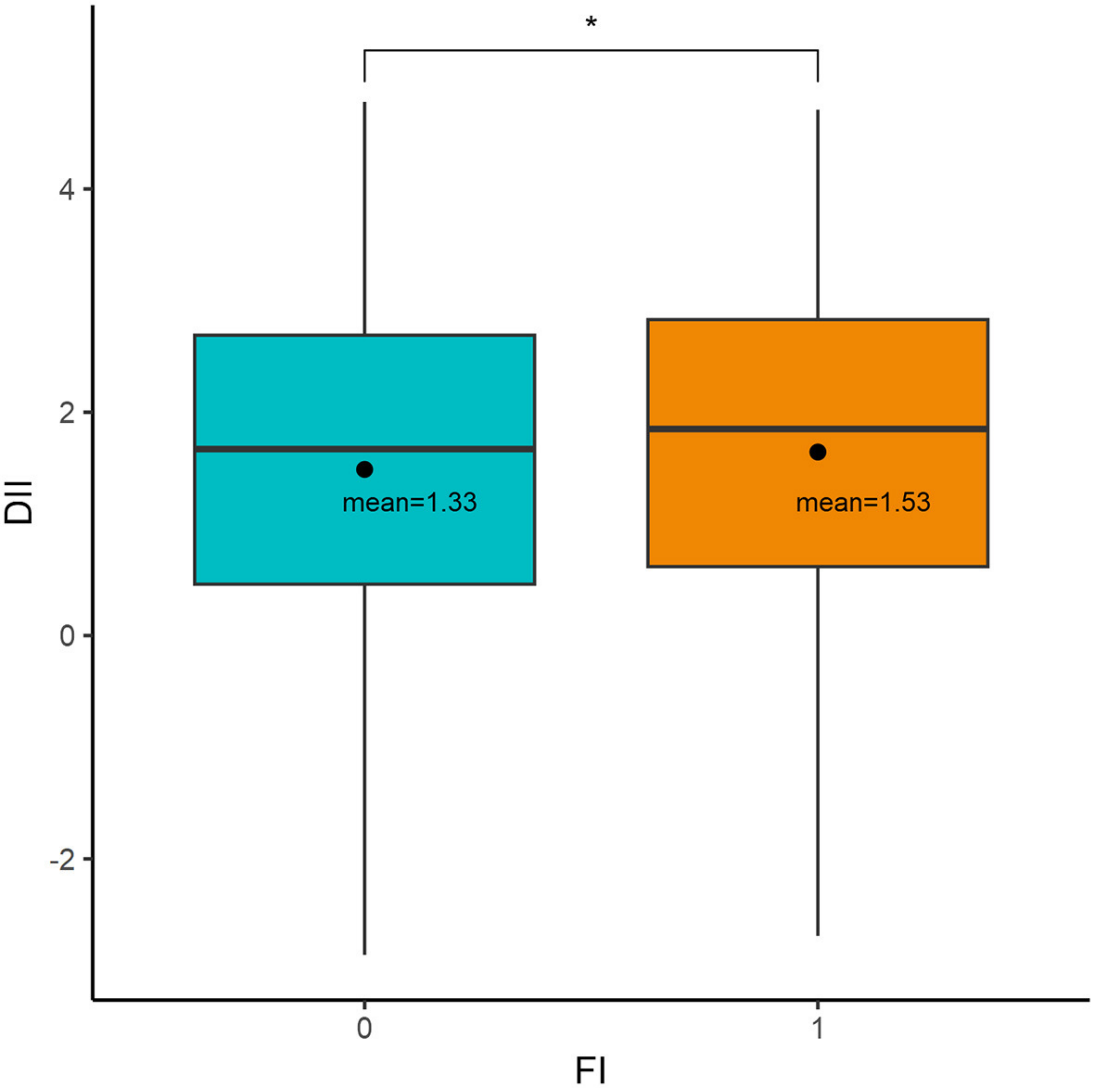
Comparison of DII between normal and participants with FI (*represents *p* < 0.05).

Table 1 summarizes the baseline characteristics of normal participants and patients with FI. Among a total of 11,747 participants, 1,068 (9.1%) experienced FI, representing 15,388,044 people. Significant differences were found between the two groups in sex, age, race, BMI, CRP, hyperlipidemia, diabetes, hypertension, and physical activity (*p* < 0.05). However, there were no statistically significant differences in educational level, PIR, energy intake, smoking status, and alcohol consumption (*p* > 0.05). Moreover, as shown in Figure 2, patients with FI had higher DII levels compared to normal participants (weighted mean DII, 1.53 vs. 1.33, *p* = 0.016).

**Table 1:** Baseline characteristics of normal and participants with FI.

**Characteristic**	**Overall (*N* = 11,747)**	**Normal (*N* = 10,679)**	**FI (*N* =1,068)**	***p*-value**
**Sex**				0.024
Male	5,700 (47.9%)	5,236 (48.5%)	464 (41.9%)	
Female	6,047 (52.1%)	5,443 (51.5%)	604 (58.1%)	
**Age, year**				< 0.001
20–44	4,967 (47.0%)	4,739 (49.2%)	228 (22.9%)	
45–64	3,979 (36.2%)	3,561 (35.3%)	418 (46.1%)	
65–85	2,801 (16.8%)	2,379 (15.5%)	422 (31.0%)	
**Race**				0.016
non-Hispanic white	6,011 (71.9%)	5,377 (71.4%)	634 (77.4%)	
non-Hispanic black	2,192 (10.5%)	2,013 (10.7%)	179 (9.1%)	
Mexican American	2,127 (8.2%)	1,982 (8.4%)	145 (5.8%)	
Other Race	1,417 (9.4%)	1,307 (NA%)	110 (NA%)	
**Educational level**				0.072
Less than high school	3,172 (17.4%)	2,830 (17.1%)	342 (21.3%)	
High school	2,804 (24.1%)	2,568 (24.3%)	236 (22.2%)	
College and above	5,771 (58.4%)	5,281 (58.6%)	490 (56.5%)	
**PIR**				0.106
< 2	4,902 (30.4%)	4,430 (30.2%)	472 (32.3%)	
≥2	6,038 (64.3%)	5,535 (64.6%)	503 (61.0%)	
Unknown	807 (5.3%)	714 (5.2%)	93 (6.7%)	
**BMI, kg/m** ^2^				0.006
< 25	3,322 (31.1%)	3,052 (31.5%)	270 (27.5%)	
25–30	4,050 (33.7%)	3,709 (34.0%)	341 (30.2%)	
≥30	4,375 (35.2%)	3,918 (34.6%)	457 (42.3%)	
CRP, mg/dL	0.4 (±0.0)	0.4 (±0.0)	0.5 (±0.1)	< 0.001
Energy intake, kcal	4,239.8 (±31.7)	4,254.2 (±33.6)	4,079.3 (±68.1)	0.063
**Smoking status**				0.090
No smoking	6,204 (53.2%)	5,709 (53.6%)	495 (48.3%)	
Past smoking	3,048 (25.0%)	2,708 (24.7%)	340 (29.1%)	
Current smoking	2,495 (21.8%)	2,262 (21.7%)	233 (22.7%)	
**Alcohol consumption**				0.090
No drinking or Light drinking	7,480 (64.5%)	6,835 (64.9%)	645 (59.3%)	
Moderate drinking	2,323 (21.4%)	2,098 (21.2%)	225 (23.4%)	
Heavy drinking	361 (3.4%)	320 (3.3%)	41 (4.1%)	
Unknown	1,583 (10.8%)	1,426 (10.6%)	157 (13.2%)	
**Hyperlipidemia**				0.006
No	7,865 (70.0%)	7,186 (70.4%)	679 (64.8%)	
Yes	3,882 (30.0%)	3,493 (29.6%)	389 (35.2%)	
**Diabetes**				< 0.001
No	10,353 (91.8%)	9,507 (92.4%)	846 (84.6%)	
Yes	1,394 (8.2%)	1,172 (7.6%)	222 (15.4%)	
**Hypertension**				< 0.001
No	7,729 (70.2%)	7,190 (71.7%)	539 (53.5%)	
Yes	4,018 (29.8%)	3,489 (28.3%)	529 (46.5%)	
**Physical activity**				< 0.001
Inactive	5,754 (41.3%)	5,099 (40.2%)	655 (53.1%)	
Moderate	3,300 (29.7%)	3,007 (29.4%)	293 (33.6%)	
Vigorous	2,693 (29.0%)	2,573 (30.4%)	120 (13.3%)	
DII	1.35 (±0.04)	1.33 (±0.04)	1.53 (±0.08)	0.016

Mean (±SE) for continuous; n (%) for categorical.

FI, fecal Incontinence; PIR, ratio of family income to poverty; BMI, body mass index; DII, dietary inflammatory index.

### The relationship between DII and FI

**Figure 3:**
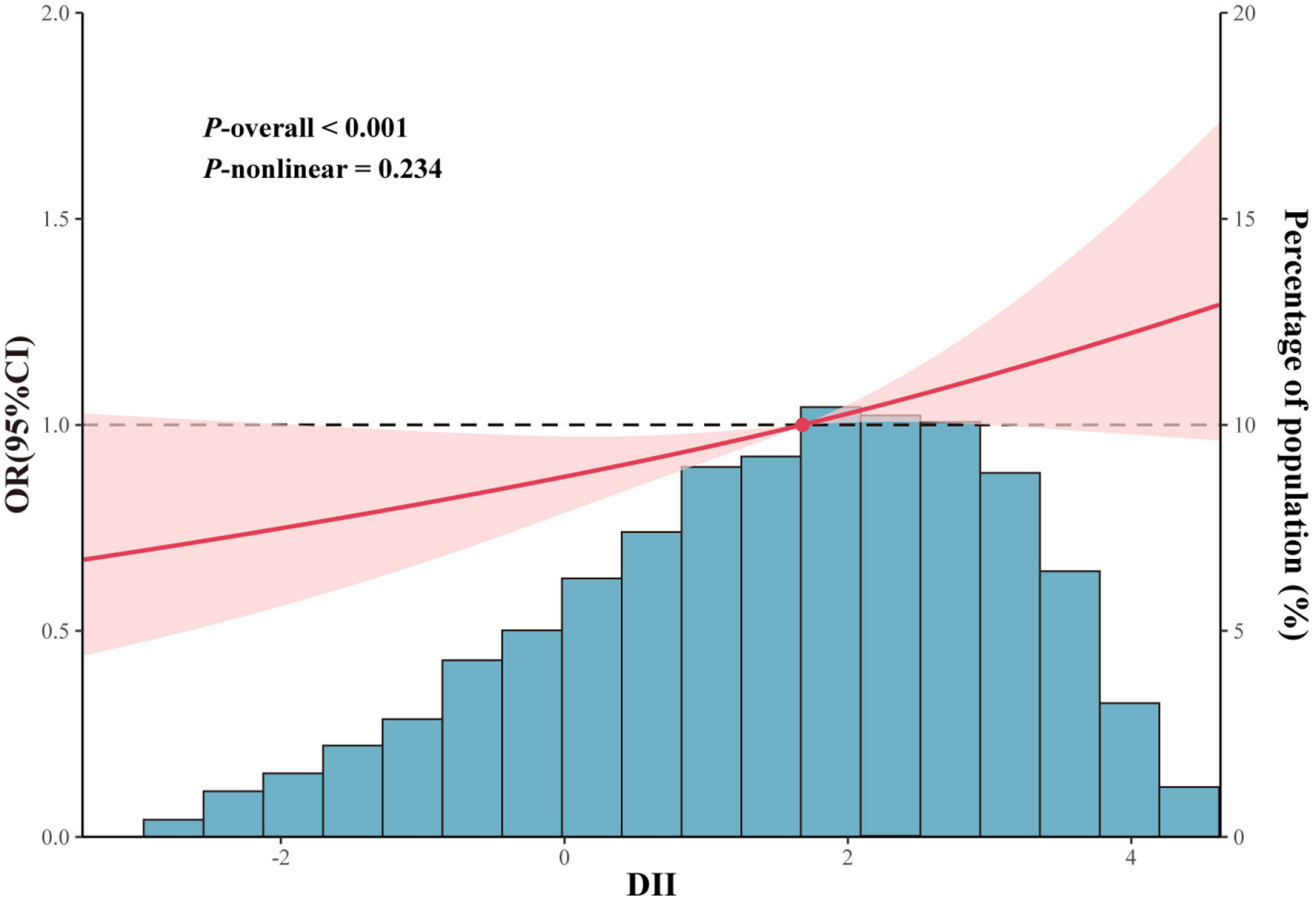
Restricted cubic spline curves between DII and FI, The DII value considered for this analysis was 1.7. The cubic spline model curves have four knots. Adjustments included sex, age, race, educational level, PIR, energy intake, BMI, hyperlipidemia, smoking status, alcohol consumption, diabetes, hypertension, physical activity, and CRP were adjusted.

Logistic regression of DII as a continuous variable found that DII was significantly correlated with FI (Crude model: OR = 1.08, 95% CI: 1.02–1.14, *p* = 0.007; Model 1: OR = 1.08, 95% CI: 1.02–1.15, *p* = 0.010; Model 2: OR = 1.11, 95% CI: 1.01–1.21, *p* = 0.032; Table 2). After DII was converted into categorical variable, DII maintained a significant correlation with FI (Q4 vs. Q1: OR = 1.40, 95% CI: 1.11–1.76, *p* = 0.005, *p* for trend = 0.011). Moreover, this correlation remains after adjustment for model 1 (Q4 vs. Q1: OR = 1.38, 95% CI: 1.11–1.73, *p* = 0.006, *p* for trend = 0.014) and model 2 (Q4 vs. Q1: OR = 1.49, 95% CI: 1.04–2.14, *p* = 0.032, *p* for trend = 0.039). The RCS curve (Figure 3) revealed that there was no non-linear correlation between DII and FI (*p*-non-linear = 0.234, *p*-overall < 0.001).

**Table 2:** The associations of DII and FI.

	**Crude model**		**Model 1**		**Model 2**	
	**OR (95% CI)**	***P***	**OR (95% CI)**	***P***	**OR (95% CI)**	***P***
Continuous	1.08 (1.02, 1.14)	0.007	1.08 (1.02, 1.15)	0.010	1.11 (1.01, 1.21)	0.032
Q1	Ref		Ref		Ref	
Q2	1.04 (0.81, 1.34)	0.734	1.08 (0.84, 1.39)	0.557	1.07 (0.82, 1.40)	0.603
Q3	1.09 (0.83, 1.42)	0.543	1.12 (0.84, 1.50)	0.426	1.17 (0.85, 1.60)	0.318
Q4	1.40 (1.11, 1.76)	0.005	1.38 (1.11, 1.73)	0.006	1.49 (1.04, 2.14)	0.032
*p* for trend		0.011		0.014		0.039

Crude model: unadjusted model; Model 1: sex, age, race, educational level, and PIR were adjusted; Model 2: sex, age, race, educational level, PIR, BMI, smoking status, alcohol consumption, physical activity, hyperlipidemia, hypertension, and diabetes, CRP, and energy intake were adjusted. DII: Q1, < 0.47; Q2, 0.47–1.68; Q3, 1.69–2.71; Q4, >2.71.

### Subgroup analyses

The results of the age subgroup analyses indicated that DII was associated with FI only in middle-aged individuals aged 45–64 years (crude model: Q3 vs. Q1, OR = 1.60, 95% CI: 1.04–2.44, *p* = 0.032). However, this significance was no longer evident after adjusting for covariates in models 1 and 2. After adjusting for all covariates, a correlation was observed between DII and FI in the subgroup aged 65–85 (model 2: Q4 vs. Q1, OR = 1.84, 95% CI: 1.07–3.18, *p* = 0.030; Supplementary Table 1).

In gender-based subgroup analyses, there was a positive association between DII and FI in the female population (crude model: Q4 vs. Q1, OR = 1.53, 95% CI: 1.05–2.24, *p* = 0.029). After adjusting for confounders, DII remained significantly associated with FI (model 1: Q4 vs. Q1, OR = 1.73, 95% CI: 1.19–2.52, *p* = 0.006; model 2: Q4 vs. Q1, OR = 2.02, 95% CI: 1.23–3.33, *p* = 0.008; Supplementary Table 2).

In the subgroup analysis stratified by race, DII was only significantly associated with FI in the non-Hispanic white group (model 2: Q4 vs. Q1: OR = 1.70, 95% CI: 1.12–2.59, *p* = 0.015), but there was no statistically significant association in the other three racial subgroups (Supplementary Table 3).

The subgroup analysis of BMI revealed an inverse relationship between DII and FI in individuals with a normal weight (model 2: Q2 vs. Q1, OR = 0.58, 95% CI: 0.35–0.97, *p* = 0.039). Conversely, as DII increased, a positive association between DII and FI was observed, although this was not statistically significant. As with the 45–60 age subgroup, DII was associated with FI in the crude model in the overweight population, and this relationship disappeared after adjusting for confounding variables. No statistical association between DII and FI was found in the obese population (Supplementary Table 4).

**Figure 4:**
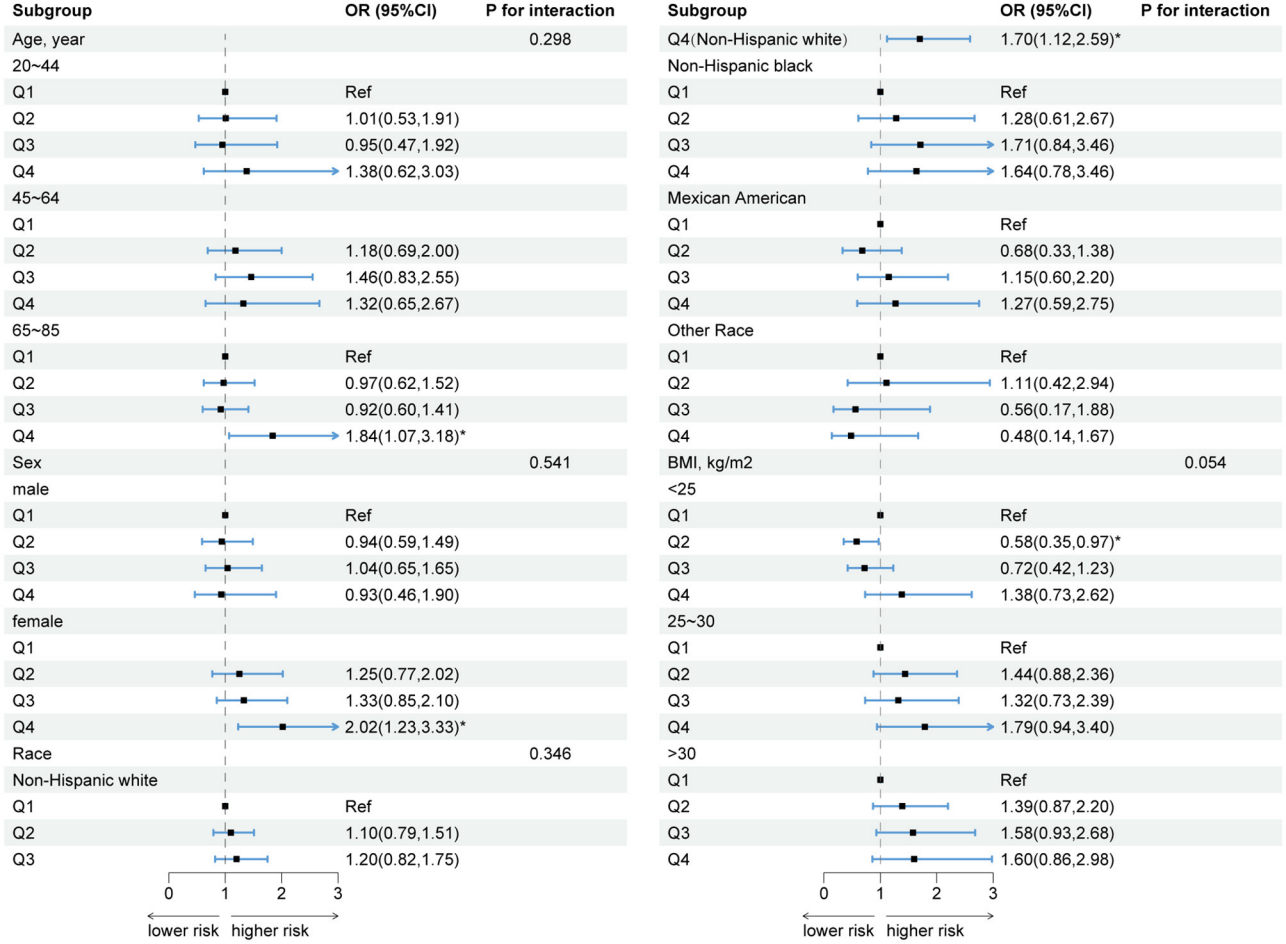
Forest plot of subgroup analyses of the relationship between DII and FI. Model 2: sex, age, race, educational level, PIR, energy intake, BMI, hyperlipidemia, smoking, alcohol consumption, diabetes, hypertension, physical activity, and CRP were adjusted. *p < 0.05.

Based on the results of the subgroup analyses, we summarized the results of model 2 and presented them in a forest plot (Figure 4). It is evident that DII is significantly associated with FI in old, female, non-Hispanic white people. In addition, no significant interaction terms were found in this study (all *p* for interaction > 0.05).

## Discussion

In this cross-sectional study conducted in the NHANES 2005–2010 survey population, we found that DII was positively associated with the occurrence of FI, a finding that persisted after adjustment for multiple confounders (OR = 1.49, *p* = 0.032). Furthermore, the dose-response curve showed no non-linear relationship between DII and FI (*p* for non-linear = 0.234). DII was substantially linked to FI in old, female, and non-Hispanic white populations.

Previous studies have examined the relationship between dietary patterns and FI but did not consider the levels of dietary inflammation. For instance, a single-arm, pre-post controlled prospective study of 39 elderly women with FI found that patients' mean Vaizey score (a scale of FI severity) decreased by 2.3 points (*p* = 0.01) after the administration of a structured dietary intervention, and 29 (74.4%) patients perceived an improvement in symptoms (29). In addition, based on previous research, Menees et al. recruited 43 patients with FI and compared the efficacy of a low FODMAP diet (LFD) for 4 weeks with psyllium 6 g/d. The results indicated that there was no statistically significant difference in the proportion of treatment responses (>50% reduction in FI episodes vs. baseline) between the two groups (38.9% for the LFD and 50% for psyllium, *p* = 0.33). Nevertheless, significant improvement in fecal incontinence was observed in the LFD group but not in the psyllium group (10). A recent large prospective study demonstrated an increased risk of fecal incontinence with a pro-inflammatory diet in older women (HR = 1.17; 95% CI, 1.08–1.27; *p* trend < 0.0001) (11), but the conclusions of this study were limited by the population of older women as well as the limited number of foods available to assess dietary inflammation. Based on a NHANES study of 14,329 individuals, Peng et al. revealed that increased DII was significantly associated with higher prevalence of abnormal bowel health and major chronic diseases, as well as higher risk of death (DII and chronic constipation: Q4 vs. Q1: OR = 1.671, 95% CI: 1.332–2.097; DII and chronic diarrhea: Q4 vs. Q1: OR = 1.484, 95% CI: 1.079–2.041; DII and all-cause mortality: Q4 vs. Q1: OR = 1.166, 95% CI: 0.947–1.434) (23). However, as a common gastrointestinal disorder, the relationship between FI and DII has not been elaborated. In our study, we demonstrated that DII was significantly associated with FI and that this same relationship held true in old, female, and non-Hispanic white populations.

It's pertinent to highlight that the underlying mechanisms that could explain our findings are still unclear. They may be related to the following. On one hand, pro-inflammatory diets can elicit gastrointestinal inflammatory responses and drive neurosensory deficits by affecting gut microbiome characteristics, leading to FI (30, 31). On the other hand, chronic inflammation caused by a pro-inflammatory diet also leads to reduced muscle mass and function and pelvic floor dysfunction, both of which are proven risk factors for FI (32, 33). In addition, the type of stool is influenced by different pathophysiological factors, such that liquid stools may be more associated with chronic intestinal disturbances, whereas solid stools are influenced by mechanical factors (32, 33). A pro-inflammatory diet may contribute to liquid stool FI by interacting with the gut microbiota and triggering subsequent intestinal disturbances (30, 32). Whereas, in the case of solid fecal FI, a diet that promotes inflammation may directly affect neuromuscular control, as inflammation may impair neuromuscular function and pelvic floor integrity (34).

Indeed, we reviewed Menees SB's research and interestingly found that the overall inflammatory effect score of carbohydrates (FLD) was only 0.097 while fibers (psyllium) was even −0.663 (anti-inflammatory effect), much lower than fat (0.298), cholesterol (0.110), saturated fatty acids (0.373), and other daily dietary components (14). In addition, fiber intake has been demonstrated to be associated with improved parameters of the inflammatory response (35), and high consumption of fruits, vegetables, whole grains, and healthy fats has been implicated in a reduced inflammatory response (36). This may also explain why a low LFD diet with fiber intake helps improve FI from a dietary inflammatory perspective.

In summary, we examined the relationship between DII and FI among adults in the US based on NHANES 2005–2010. FI is a common intestinal dysfunction associated with a poor prognosis. Our study suggests that a high DII diet increases the risk of developing FI, particularly in old, female, and non-Hispanic white populations. This finding may assist in providing dietary strategies and therapeutic advice to patients with FI in the clinical setting. For people with FI, reducing the intake of inflammatory foods, such as saturated fat, trans fat, total fat, and cholesterol, may be a good dietary strategy. However, the specific mechanism regarding DII leading to FI is not clear and needs to be investigated in a step study.

This study has unique strengths. First, it is a survey study based on national population data with a large sample size. Second, the scientific sampling design of NHANES and the comprehensive health and nutritional data allowed us to adjust for many potential confounders, which improved the reliability of the results. However, we have to admit that there are some inherent limitations. Firstly, it was a cross-sectional study, which precludes us from establishing a causal relationship between DII and FI. Secondly, the study population was adults aged 20 years or older, posing that the findings may not generalize to those younger than 20 years. Thirdly, the absence of data precluded our ability to exclude the influence of numerous other significant potential confounding variables associated with FI. Lastly, data on FI was obtained from patient recollections during interviews, which may be subject to recall bias when compared with the records of prospective experiments (37).

## Conclusion

DII is significantly and positively associated with FI, especially in old, female, and non-Hispanic white individuals. Reducing daily dietary inflammatory level may be an effective strategy to prevent FI, but the exact mechanisms need to be further explored.

## Data availability statement

The original contributions presented in the study are included in the article/Supplementary material, further inquiries can be directed to the corresponding author.

## Ethics statement

The studies involving humans were approved by National Center for Health Statistics Ethics Review Board. The studies were conducted in accordance with the local legislation and institutional requirements. The participants provided their written informed consent to participate in this study.

## Author contributions

ZL: Data curation, Formal analysis, Investigation, Methodology, Software, Supervision, Validation, Visualization, Writing – original draft, Writing – review & editing. XC: Data curation, Formal analysis, Investigation, Methodology, Validation, Writing – original draft. JH: Resources, Software, Supervision, Validation, Visualization, Writing – review & editing. FC: Funding acquisition, Investigation, Methodology, Writing – review & editing. ZW: Funding acquisition, Investigation, Methodology, Writing – review & editing. LY: Investigation, Methodology, Project administration, Writing – review & editing. XL: Software, Supervision, Validation, Writing – review & editing. WS: Conceptualization, Formal analysis, Project administration, Supervision, Writing – review & editing.
