# Supplementary material for: Association between dietary inflammatory index and fecal incontinence in American adults: a cross-sectional study from NHANES 2005–2010
Source: Front Nutr. 2024 Jul 15;11:1364835. doi: 10.3389/fnut.2024.1364835 (PMC11284164; doi:10.3389/fnut.2024.1364835)
Supplement: Supplementary file 1 [file Table_1.docx]

Table S1. Subgroup analyses between DII and FI based on age.

| **Age, year** | **Crude model** | |  | **Model 1** | |  | **Model 2** | |
| --- | --- | --- | --- | --- | --- | --- | --- | --- |
|  | **95%CI** | **P** |  | **95%CI** | **P** |  | **95%CI** | **P** |
| **20-44** |  |  |  |  |  |  |  |  |
| Q1 | ref |  |  | ref |  |  | ref |  |
| Q2 | 1.12(0.66,1.89) | 0.667 |  | 1.08(0.63,1.86) | 0.780 |  | 1.01(0.53,1.91) | 0.983 |
| Q3 | 1.02(0.60,1.74) | 0.948 |  | 0.98(0.56,1.72) | 0.940 |  | 0.95(0.47,1.92) | 0.874 |
| Q4 | 1.57(0.89,2.77) | 0.118 |  | 1.49(0.82,2.68) | 0.183 |  | 1.38(0.62,3.03) | 0.411 |
| p for trend |  | 0.186 |  |  | 0.26 |  |  | 0.457 |
| **45-64** |  |  |  |  |  |  |  |  |
| Q1 | ref |  |  | ref |  |  | ref |  |
| Q2 | 1.27(0.80,2.01) | 0.300 |  | 1.22(0.78,1.92) | 0.366 |  | 1.18(0.69,2.00) | 0.533 |
| Q3 | 1.60(1.04,2.44) | 0.032 |  | 1.50(0.96,2.32) | 0.071 |  | 1.46(0.83,2.55) | 0.180 |
| Q4 | 1.52(0.99,2.35) | 0.057 |  | 1.33(0.85,2.09) | 0.205 |  | 1.32(0.65,2.67) | 0.421 |
| p for trend |  | 0.024 |  |  | 0.119 |  |  | 0.32 |
| **65-85** |  |  |  |  |  |  |  |  |
| Q1 | ref |  |  | ref |  |  | ref |  |
| Q2 | 0.85(0.54,1.34) | 0.471 |  | 0.91(0.58,1.42) | 0.667 |  | 0.97(0.62,1.52) | 0.892 |
| Q3 | 0.74(0.49,1.11) | 0.140 |  | 0.79(0.53,1.18) | 0.241 |  | 0.92(0.60,1.41) | 0.703 |
| Q4 | 1.31(0.84,2.05) | 0.229 |  | 1.46(0.94,2.26) | 0.092 |  | 1.84(1.07,3.18) | 0.030 |
| p for trend |  | 0.331 |  |  | 0.157 |  |  | 0.051 |

Note: Crude Model: Unadjusted model; Model 1: sex, age, educational level, and PIR were adjusted; Model2: sex, age, race, educational level, PIR, BMI, smoking status, alcohol consumption, physical activity, hyperlipidemia, hypertension, and diabetes, CRP, and energy intake were adjusted. DII: Q1, <0.47; Q2, 0.47-1.68; Q3, 1.69-2.71; Q4, >2.71.

Table S2. Subgroup analyses between DII and FI based on sex.

| **Sex** | **Crude model** | |  | **Model 1** | |  | **Model 2** | |
| --- | --- | --- | --- | --- | --- | --- | --- | --- |
|  | **OR(95%CI)** | **P** |  | **OR(95%CI)** | **P** |  | **OR(95%CI)** | **P** |
| **Male** |  |  |  |  |  |  |  |  |
| Q1 | ref |  |  | ref |  |  | ref |  |
| Q2 | 0.93(0.62,1.38) | 0.702 |  | 0.97(0.65,1.44) | 0.864 |  | 0.94(0.59,1.49) | 0.770 |
| Q3 | 1.06(0.69,1.64) | 0.784 |  | 1.10(0.69,1.75) | 0.687 |  | 1.04(0.65,1.65) | 0.876 |
| Q4 | 1.01(0.67,1.54) | 0.952 |  | 0.99(0.64,1.53) | 0.955 |  | 0.93(0.46,1.90) | 0.836 |
| p for trend |  | 0.81 |  |  | 0.844 |  |  | 0.93 |
| **Female** |  |  |  |  |  |  |  |  |
| Q1 | ref |  |  | ref |  |  | ref |  |
| Q2 | 1.16(0.72,1.85) | 0.541 |  | 1.24(0.78,1.98) | 0.358 |  | 1.25(0.77,2.02) | 0.348 |
| Q3 | 1.08(0.71,1.62) | 0.717 |  | 1.22(0.81,1.83) | 0.330 |  | 1.33(0.85,2.10) | 0.202 |
| Q4 | 1.53(1.05,2.24) | 0.029 |  | 1.73(1.19,2.52) | 0.006 |  | 2.02(1.23,3.33) | 0.008 |
| p for trend |  | 0.033 |  |  | 0.01 |  |  | 0.01 |

Note: Crude Model: unadjusted model; Model 1: sex, age, educational level, and PIR were adjusted; Model2: sex, age, race, educational level, PIR, BMI, smoking status, alcohol consumption, physical activity, hyperlipidemia, hypertension, and diabetes, CRP, and energy intake were adjusted. DII: Q1, <0.47; Q2, 0.47-1.68; Q3, 1.69-2.71; Q4, >2.71.

Table S3. Subgroup analyses between DII and FI based on race.

| **Race** | **Crude model** | |  | **Model 1** | |  | **Model 2** | |
| --- | --- | --- | --- | --- | --- | --- | --- | --- |
|  | **OR(95%CI)** | **P** |  | **OR(95%CI)** | **P** |  | **OR(95%CI)** | **P** |
| **non-Hispanic white** |  |  |  |  |  |  |  |  |
| Q1 | ref |  |  | ref |  |  | ref |  |
| Q2 | 1.08(0.81,1.43) | 0.611 |  | 1.09(0.80,1.49) | 0.557 |  | 1.10(0.79,1.51) | 0.563 |
| Q3 | 1.13(0.81,1.59) | 0.457 |  | 1.13(0.78,1.65) | 0.501 |  | 1.20(0.82,1.75) | 0.338 |
| Q4 | 1.62(1.25,2.10) | <0.001 |  | 1.55(1.19,2.03) | 0.002 |  | 1.70(1.12,2.59) | 0.015 |
| p for trend |  | 0.002 |  |  | 0.008 |  |  | 0.022 |
| **non-Hispanic black** |  |  |  |  |  |  |  |  |
| Q1 | ref |  |  | ref |  |  | ref |  |
| Q2 | 1.10(0.59,2.04) | 0.763 |  | 1.20(0.63,2.31) | 0.571 |  | 1.28(0.61,2.67) | 0.496 |
| Q3 | 1.36(0.73,2.53) | 0.320 |  | 1.52(0.77,3.01) | 0.221 |  | 1.71(0.84,3.46) | 0.132 |
| Q4 | 1.22(0.70,2.13) | 0.483 |  | 1.28(0.70,2.35) | 0.410 |  | 1.64(0.78,3.46) | 0.182 |
| p for trend |  | 0.354 |  |  | 0.342 |  |  | 0.1 |
| **Mexican American** |  |  |  |  |  |  |  |  |
| Q1 | ref |  |  | ref |  |  | ref |  |
| Q2 | 0.53(0.25,1.15) | 0.105 |  | 0.51(0.23,1.10) | 0.084 |  | 0.68(0.33,1.38) | 0.270 |
| Q3 | 0.76(0.44,1.32) | 0.322 |  | 0.70(0.39,1.24) | 0.217 |  | 1.15(0.60,2.20) | 0.665 |
| Q4 | 0.77(0.40,1.49) | 0.433 |  | 0.70(0.36,1.36) | 0.287 |  | 1.27(0.59,2.75) | 0.529 |
| p for trend |  | 0.622 |  |  | 0.431 |  |  | 0.303 |
| **Other race** |  |  |  |  |  |  |  |  |
| Q1 | ref |  |  | ref |  |  | ref |  |
| Q2 | 1.39(0.60,3.22) | 0.428 |  | 1.33(0.57,3.14) | 0.503 |  | 1.11(0.42,2.94) | 0.829 |
| Q3 | 0.90(0.31,2.60) | 0.844 |  | 0.86(0.31,2.38) | 0.773 |  | 0.56(0.17,1.88) | 0.337 |
| Q4 | 0.94(0.33,2.69) | 0.905 |  | 0.81(0.28,2.37) | 0.699 |  | 0.48(0.14,1.67) | 0.236 |
| p for trend |  | 0.731 |  |  | 0.535 |  |  | 0.139 |

Note: Crude Model: unadjusted model; Model 1: sex, age, educational level, and PIR were adjusted; Model2: sex, age, race, educational level, PIR, BMI, smoking status, alcohol consumption, physical activity, hyperlipidemia, hypertension, and diabetes, CRP, and energy intake were adjusted. DII: Q1, <0.47; Q2, 0.47-1.68; Q3, 1.69-2.71; Q4, >2.71.

Table S4. Subgroup analyses between DII and FI based on BMI.

| BMI, kg/m^2^ | Crude model | |  | Model 1 | |  | Model 2 | |
| --- | --- | --- | --- | --- | --- | --- | --- | --- |
|  | OR(95%CI) | P |  | OR(95%CI) | P |  | OR(95%CI) | P |
| <25 |  |  |  |  |  |  |  |  |
| Q1 | ref |  |  | ref |  |  | ref |  |
| Q2 | 0.57(0.33,0.98) | 0.042 |  | 0.59(0.35,1.00) | 0.048 |  | 0.58(0.35,0.97) | 0.039 |
| Q3 | 0.67(0.42,1.07) | 0.093 |  | 0.73(0.46,1.17) | 0.185 |  | 0.72(0.42,1.23) | 0.222 |
| Q4 | 1.32(0.79,2.20) | 0.277 |  | 1.45(0.87,2.40) | 0.147 |  | 1.38(0.73,2.62) | 0.306 |
| p for trend |  | 0.426 |  |  | 0.248 |  |  | 0.395 |
| 25-30 |  |  |  |  |  |  |  |  |
| Q1 | ref |  |  | ref |  |  | ref |  |
| Q2 | 1.36(0.90,2.04) | 0.136 |  | 1.36(0.87,2.13) | 0.169 |  | 1.44(0.88,2.36) | 0.137 |
| Q3 | 1.22(0.81,1.85) | 0.326 |  | 1.18(0.72,1.93) | 0.498 |  | 1.32(0.73,2.39) | 0.335 |
| Q4 | 1.62(1.05,2.49) | **0.030** |  | 1.50(0.90,2.52) | 0.118 |  | 1.79(0.94,3.40) | 0.073 |
| p for trend |  | 0.06 |  |  | 0.209 |  |  | 0.123 |
| >30 |  |  |  |  |  |  |  |  |
| Q1 | ref |  |  | ref |  |  | ref |  |
| Q2 | 1.17(0.76,1.81) | 0.458 |  | 1.23(0.79,1.91) | 0.343 |  | 1.39(0.87,2.20) | 0.158 |
| Q3 | 1.32(0.81,2.15) | 0.265 |  | 1.38(0.82,2.32) | 0.212 |  | 1.58(0.93,2.68) | 0.090 |
| Q4 | 1.27(0.78,2.05) | 0.324 |  | 1.24(0.77,1.99) | 0.374 |  | 1.60(0.86,2.98) | 0.132 |
| p for trend |  | 0.283 |  |  | 0.34 |  |  | 0.127 |

Note: Crude Model: unadjusted model; Model 1: sex, age, educational level, and PIR were adjusted; Model2: sex, age, race, educational level, PIR, BMI, smoking status, alcohol consumption, physical activity, hyperlipidemia, hypertension, and diabetes, CRP, and energy intake were adjusted. DII: Q1, <0.47; Q2, 0.47-1.68; Q3, 1.69-2.71; Q4, >2.71.
